# Suppression of head and neck cancer cell survival and cisplatin resistance by GRP78 small molecule inhibitor YUM70

**DOI:** 10.3389/fonc.2022.1044699

**Published:** 2023-01-11

**Authors:** Vicky Yamamoto, Bintao Wang, Amy S. Lee

**Affiliations:** ^1^ Department of Biochemistry and Molecular Medicine, University of Southern California, Keck School of Medicine, Los Angeles, CA, United States; ^2^ USC Norris Comprehensive Cancer Center, Los Angeles, CA, United States

**Keywords:** GRP78, head and neck (H&N) cancer, cisplatin resistance, YUM70, apoptosis, cell viability, spheroid, head and neck squamous cell carcinoma

## Abstract

**Background:**

Head and neck squamous cell carcinoma (HNSCC) is one of the leading causes of cancer-related death worldwide. Surgical resection, radiation and chemotherapy are the mainstay of HNSCC treatment but are often unsatisfactory. Cisplatin is a commonly used chemotherapy in HNSCC; however, cisplatin resistance is a major cause of relapse and death. The 78-kD glucose-regulated protein (GRP78) is the master regulator of the unfolded protein response (UPR) and is implicated in therapeutic resistance in cancer. The role of GRP78 in cisplatin resistance in HNSCC remains unclear. YUM70 is a newly discovered hydroxyquinoline analogue and found to be an inhibitor of GRP78. The effect of YUM70 in HNSCC cell lines is unknown.

**Method:**

Knockdown of GRP78 by siRNAs was performed to investigate the effect of GRP78 reduction in endoplasmic reticulum (ER)-stress induced and general apoptosis. Western blots examining apoptotic markers were performed on three HPV-negative HNSCC cell lines. WST-1 assay was performed to determine cell viability. In reverse, we utilized AA147, an ER proteostasis regulator to upregulate GRP78, and apoptotic markers and cell viability were determined. To test the ability of YUM70 to reverse cisplatin resistance, cisplatin-resistant HNSCC cell lines were generated by prolonged, repeated exposure to increasing concentrations of cisplatin. Colony formation assay using the cisplatin-resistant HNSCC cell line was performed to assess the *in vitro* reproductive cell survival. Furthermore, to test the ability of YUM70 to reverse cisplatin resistance in a physiologically relevant system, we subjected the 3D spheroids of the cisplatin-resistant HNSCC cell line to cisplatin treatment with or without YUM70 and monitored the onset of apoptosis.

**Results:**

Reduction of GRP78 level induced HNSCC cell death while GRP78 upregulation conferred higher resistance to cisplatin. Combined cisplatin and YUM70 treatment increased apoptotic markers in the cisplatin-resistant HNSCC cell line, associating with reduced cell viability and clonogenicity. The combination treatment also increased apoptotic markers in the 3D spheroid model.

**Conclusion:**

The GRP78 inhibitor YUM70 reduced HNSCC cell viability and re-sensitized cisplatin-resistant HNSCC cell line in both 2D and 3D spheroid models, suggesting the potential use of YUM70 in the treatment of HNSCC, including cisplatin-resistant HNSCC.

## Introduction

Globally, head and neck cancer (HNC) is the seventh most common cancer with an annual incidence of over 740,000 new cases and 360,000 deaths reported (1). Thus, there is an urgent need for discoveries of new therapeutic agents for treatment of HNC. Approximately 90% of HNC is classified as head and neck squamous cell carcinoma (HNSCC). Primary causes of HNSCC include tobacco smoking, heavy alcohol consumption, and human papillomavirus (HPV). While HPV-negative HNSCC arises in any regions in head and neck, HPV-positive HNSCC arises almost exclusively in oropharynx (2, 3). While the main treatment modalities are surgery, radiation therapy and chemotherapy, recent advances include trans-oral robotic surgery (TORS), FDA approval of immunotherapy drugs as well as cetuximab, a targeted therapy available for HNSCC (4–6). However, overall survival has not been improved significantly in the last four decades, especially in the advanced-stage, HPV-negative HNSCC (7, 8). Recurrent and metastatic HNSCC are treatment resistance and thus largely incurable, which are the major causes of the treatment failure and death in HNSCC (9, 10). Platinum-based chemotherapy drugs, such as cisplatin, are the most commonly used chemotherapeutics in HNSCC; however, originally sensitive tumors frequently acquire resistance eventually, which hampers the success of treatment (11). The mechanism(s) of cisplatin resistance can be multifaceted and multifactorial. Possible mechanisms of cisplatin resistance include alteration in the DNA repair mechanisms, drug efflux, apoptosis initiation/inhibition, intracellular detoxification, and metabolic reprogramming (10, 12, 13).

The unfolded protein response (UPR) is an evolutionarily conserved mechanism for cells to adapt to endoplasmic reticulum (ER) stress and the ER stress signaling has been known to confer chemotherapy resistance in solid tumors, including cisplatin resistance (14). GRP78, also referred to as BiP or HSPA5, is a key ER chaperone and a master regulator of the UPR (15, 16). As a major pro-survival component of the UPR, GRP78 has been associated with cisplatin-resistance in cervical cancer and melanoma (17, 18). In HNSCC, GRP78 has been reported to promote cell growth, migration and invasion (19), and in tumor initiating cells, maintain their stemness properties (20–22). Moreover, it has been reported that GRP78 causes treatment resistant through maintaining lysosomal activity (23). Thus, GRP78 is emerging as a biomarker and a therapeutic target for HNSCC.

GRP78 expression can be modulated by affecting its transcription, translation, stability or activity. For GRP78 upregulation, AA147 is a small molecule with low toxicity that preferentially activates the ATF6 pathway of the UPR without causing global UPR activation (24). AA147 induces transcription of GRP78, which is a major downstream gene target of ATF6 (24). On the other hand, YUM70 is a novel hydroxyquinoline that directly binds GRP78 and inactivates its enzymatic activity, resulting in ER stress-mediated apoptosis (25). This newly identified, potent GRP78 inhibitor exhibits preclinical efficacy in a pancreatic cancer xenograft model with no toxicity to normal tissues, as a monotherapy or in combinative therapy (25). Nonetheless, the efficacy of YUM70 in HNSCC remains to be determined.

Here, we report on the effects of AA147 and YUM70 on HNSCC, with the latter as a potential therapeutic agent to combat cisplatin resistance. Our results show that while upregulation of GRP78 by AA147 confers resistance to cisplatin, inhibition of GRP78 activity by YUM70 reduces HNSCC cell line viability, enhances cisplatin sensitivity, and induces cell death in cisplatin-resistant HNSCC cell lines both in 2D culture and in 3D spheroid models. These results suggest that the GRP78 inhibitor YUM70 holds promise as a therapeutic agent in the treatment of HNSCC and overcoming cisplatin resistance.

## Materials and methods

### Reagents and drugs

YUM70, kindly provided by Dr. Nouri Neamati (University of Michigan), was dissolved in dimethyl sulfoxide (DMSO). AA147, kindly provided by Dr. Luke Wiseman (Scripps Research Institute) was dissolved in DMSO. Thapsigargin (Tg) was purchased from MilliporeSigma (Burlington, MA) and dissolved in DMSO. For ER-stress induction, the cells were treated with Tg at 300 nM for 24 hr. Cisplatin (MilliporeSigma, Burlington, MA) was dissolved in saline (0.15 M sodium chloride solution). Final DMSO concentration was 0.1%. Dulbecco’s modified Eagle’s medium (DMEM), DMEM/F12 as well as fetal bovine serum (FBS), 100X penicillin-streptomycin, 0.05% trypsin-EDTA, and phosphate-buffered saline (PBS) were purchased from Corning (Glendale, AZ). 100X N2 Supplement, basic FGF (b-FGF), and EGF were purchased from ThermoFisher Scientific (Waltham, MA).

### Cell lines and culture condition

HNSCC cell lines SCC15 (tongue HNSCC), SCC25 (tongue HNSCC) were purchased from ATCC, and SCC351 (maxilla HNSCC) was a gift from Dr. Alan Epstein (26) (University of Southern California). All three cell lines were cultured in DMEM supplemented with 10% FBS and 1% penicillin/streptomycin, and maintained at 37°C in a humidified atmosphere of 95% air and 5% CO_2_. All cells were screened for mycoplasma by real-time PCR assay using primers specific for mycoplasma 16S rRNA as previously described (27).

### Generation of cisplatin-resistant cell line

To generate cisplatin-resistant HNSCC cell lines, SCC15 cells were treated with increasing doses of cisplatin for 48 hr, starting from 6 μM to 12 μM over the course of 6 months as previously described by others (28). The media were removed and cells were allowed to recover until the cells become confluent. Then the cells were re-seeded at 1:5 to 1:10 dilution and once the cells reached 50% confluency, cells were treated with cisplatin again for 48 hr and the process was repeated 25 times. Three cisR-SCC15 lines (cisR-1, cisR-2, and cisR-3) were generated.

### 3D spheroid culture

CisR-3 cells were cultured in ultra-low attachment 6-well plates (Corning, Glendale, AZ) under serum-free condition. The cells were grown in DMEM/F12 supplemented with 1% penicillin-streptomycin, 1% N2 supplement, 10 nM bFGF and 10 nM EGF. The media with the growth factors were replaced every 2 to 3 days. The spheroids were grown to 50 cells or more at which the spheroids were treated with the drugs for 48 hr then harvested for immunoblot analysis.

### TCGA analysis

GRP78 gene expression analysis in HNSCC and normal tissues was obtained from the University of Alabama at Birmingham cancer data analysis portal (UALCAN) (29) and the Gene Expression Profiling Interactive Analysis 2 (GEPIA2) (30). The normal tissues included the matched adjacent normal head and neck tissues from the same patients in the TCGA database. Different subtypes are categorized by TCGA-based gene expression (31). The high- and low-expression cohorts were separated by the median value of GRP78 expression.

### Transfection of siRNAs

Transfection of siRNAs was performed as previously described (32). Briefly, the cells were transfected with siRNAs targeting *GRP78* (si78) or control scrambled (siCtrl) using Lipofectamine RNAiMAX Tranfection Reagent (ThermoFisher Scientific, Waltham, MA). Cell lysates were harvested 48 hr after the transfection for Western blot analysis. Custom siRNAs were purchased from GE Healthcare Dharmacon, Inc. (Chicago, IL). The sequences of the siRNAs are as follow: siCtrl: 5’-GAGAUCGUAUAGCAACGGU-3’; si78 (CDS): 5’-GGAGCGCAUUGAUACUAGA-3’; si78 (3’UTR): 5’-CUUAAGUCUCGAAUGUAAU-3’.

### RNA extraction and RT-qPCR

RNA extraction and reverse transcription procedures have been described (33). The amplification protocol was set as follows: denaturation at 95°C for 10 min followed by 40 cycles of 15 seconds of denaturation at 95°C, 30 seconds of annealing/extension and data collection at 60°C. The primers for human *GRP78* are 5’-GGTGAAAGACCCCTGACAAA-3’ and 5’-GTCAGGCGATTCTGGTCATT-3’, for human *β-actin* are 5’-TCCCTGGAGAAGAGCTACGA-3’ and 5’-AGCACTGTGTTGGCGTACAG-3’. Relative mRNA abundance was calculated and normalized to the levels of *β-actin* as follows: ΔCt=Ct-genes – Ct-*β-actin* and ΔΔCt=ΔCt-sample – ΔCt-reference.

### Immunoblot analysis

Immunoblot was performed as previously described (34). The cell lysates were subjected to 10% to 12% SDS-PAGE. Primary antibodies used in this study were as follows: mouse anti-GRP78 (BiP, 1:2000, BD Transduction Laboratories #610979), mouse anti-GAPDH (1:2000, Santa Cruz #sc-32233), mouse anti-CHOP (1:1000, Cell Signaling #2895), rabbit anti-cleaved-PARP (1:1000, Cell Signaling #5625), and rabbit anti-cleaved-Caspase 7 (1:1000, Cell Signaling #8438). Secondary antibodies used were as follows: HRP-conjugated goat anti-mouse (1:5000, Santa Cruz #sc-516102), HRP-conjugated goat anti-rabbit (1:3000, Santa Cruz #sc-2357). The band quantification was determined using the Bio-Rad Image Lab 6.0.1 software.

### Cell viability assay

Cell viability was measured using the WST-1 assay following the manufacturer’s recommendations (Millipore Sigma, Burlington, MA). Briefly, HNSCC cell lines were seeded into 96-well plates at 1×10^4^ cells/well in a volume of 100 µl medium and incubated at 37°C for 16 hr. The cells were then treated with drugs for 48 hr. Cell viability was assayed using a Model 680 Microplate Reader (Bio-Rad Laboratories, Hercules, CA) at 450 nm. IC50 was determined as a drug concentration showing 50% cell growth inhibition as compared with control cell growth.

### Phase contrast cell imaging

The cells were seeded in 6 cm dishes and following treatment, the cell images at either 40X (for 3D spheroids) or 100X (for 2D cells) were captured by a Nikon TS100 inverted light microscope (Nikon Corporation, Tokyo, Japan).

### Colony formation assay

The cells were seeded at 1X10^3^ in 6-well plates. Following treatment, the media was replaced with fresh media and the cells were allowed to grow for 12 days. Cells were washed with PBS, fixed in 100% methanol followed by 0.5% crystal violet staining for visualization of surviving colonies.

### Statistical analysis

All pair-wise comparisons were made using a two-tailed unpaired Student’s *t*-test in GraphPad Prism version 5 or Microsoft Excel. *P*-values of less than 0.05 were regarded as statistically significant.

## Results

### GRP78 knockdown or inhibition of its activity by YUM70 induces apoptosis in HNSCC cell lines

**Figure 1:**
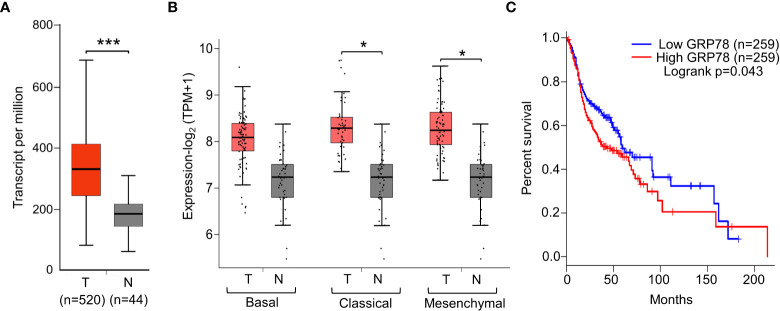
Elevated GRP78 expression in HNSCC tissues associates with poor prognosis. **(A)** Gene expression analysis of *GRP78* in HNSCC using UALCAN tool based on the TCGA database. Box plots represent the relative expression of *GRP78* in terms of transcript per million in the tumor (red, n=520) and normal (gray, n=44) samples. **(B)** Gene expression analysis of *GRP78* in sub-types of HNSCC using GEPIA2 tool based on the TCGA database. Box plots represent the *GRP78* gene expression level in terms of log_2_(TPM+1) in the tumor (red, n=87 for Basal, n=49 for Classical, n=75 for Mesenchymal) and normal (grey, n=44) samples. **(C)** Kaplan–Meier plot showing the association of GRP78 expression with HNSCC patient survival (blue line—lower expression; red line—higher expression). *P ≤ 0.05, ***P ≤ 0.001 (Student's *t* test).

First, to validate the clinical significance of GRP78, GRP78 expression level was assessed in the HNSCC patient tissues. Analysis of the UALCAN data set and the average value of GRP78 transcript level revealed that GRP78 expression is significantly higher in head and neck tumors compared to normal tissues (p<0.001) (**Figure 1A**). Genome-wide analyses performed by TCGA further revealed higher GRP78 expression levels in classical HPV-negative HNSCC, the predominant type of HNSCC, as well as mesenchymal type of HNSCC (p<0.05) (**Figure 1B**). Correspondingly, high expression of GRP78 was correlated with poor survival (p=0.043; HR=1.3, P(HR)=0.044) (**Figure 1C**).

**Figure 2:**
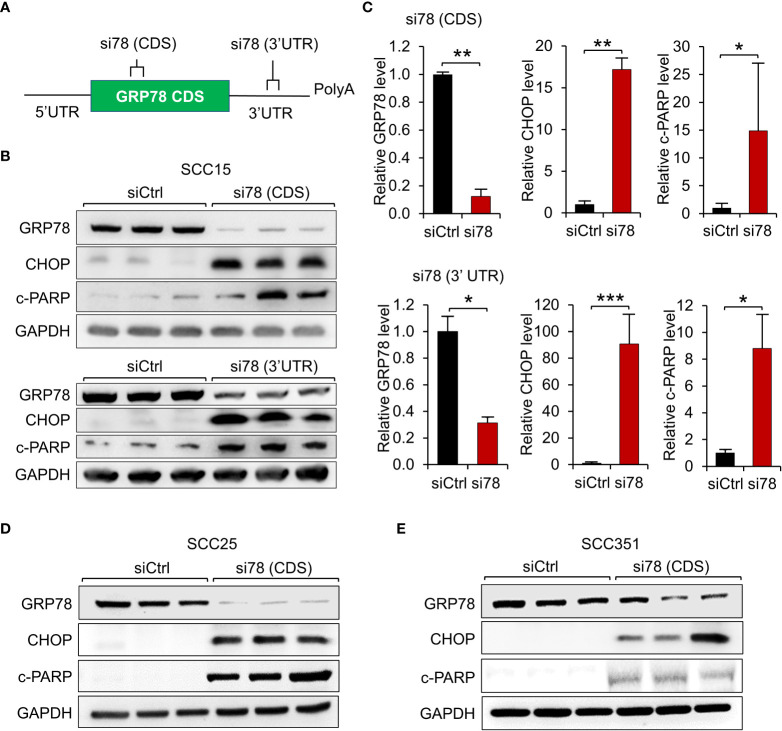
GRP78 knockdown induces apoptosis in HNSCC cell lines. **(A)** Schematic drawing of the locations of the siRNAs targeting the coding sequence (CDS) or the 3’ untranslated region (UTR) of the *GRP78* mRNA. **(B)** Western blot analysis of lysates from SCC15 cells transfected with si78 (CDS), si78 (3’UTR) or control siRNA (siCtrl) with a scrambled RNA sequence. The protein levels of GRP78, CHOP and cleaved-PARP (c-PARP) were determined, with GAPDH serving as loading control. **(C)** Quantitation of the relative levels of GRP78, CHOP and c-PARP after normalization with GAPDH serving as loading control. Data are presented as means ± S.D. (n=3). *P ≤ 0.05, **P ≤ 0.01, ***P ≤ 0.001 (Student’s *t* test). **(D)** Western blot analysis of lysates from SCC25 cells and **(E)** from SCC351 cells transfected with si78 (CDS) or control siRNA (siCtrl) for the indicated proteins.

To determine the functional contribution of GRP78 in HNSCC survival, 3 HPV-negative HNSCC cell lines (SCC15, SCC25 and SCC351) were examined. In our first approach, knockdown of GRP78 was achieved through the use of siRNAs. To rule out off-target effects of siRNA, we utilized 2 different siRNAs against *GRP78*, with one targeting the coding sequence of *GRP78* [si78 (CDS)], and the other one targeting the 3’UTR sequence of *GRP78* [si78 (3’UTR)] (**Figure 2A**). SCC15 cells, a classical HPV-negative HNSCC, were treated with either control siRNA (siCtrl), si78 (CDS) or si78 (3’UTR), and assayed for levels of GRP78, as well as general apoptotic marker (cleaved-PARP), and ER-stress induced apoptotic marker (CHOP) in Western blots. Treatment with si78 (CDS) or si78 (3’UTR) efficiently reduced the GRP78 protein level to about 10% to 30% respectively of the control siRNA group (**Figures 2B, C**). Both siRNAs against GRP78 led to significant increase in CHOP and cleaved-PARP, in agreement of GRP78 depletion resulted in the onset of ER stress-induced and general apoptosis in the HNSCC cell line (**Figures 2B, C**). Similar results were observed in SCC25 and SCC351 cells (**Figures 2D, E**).

**Figure 3:**
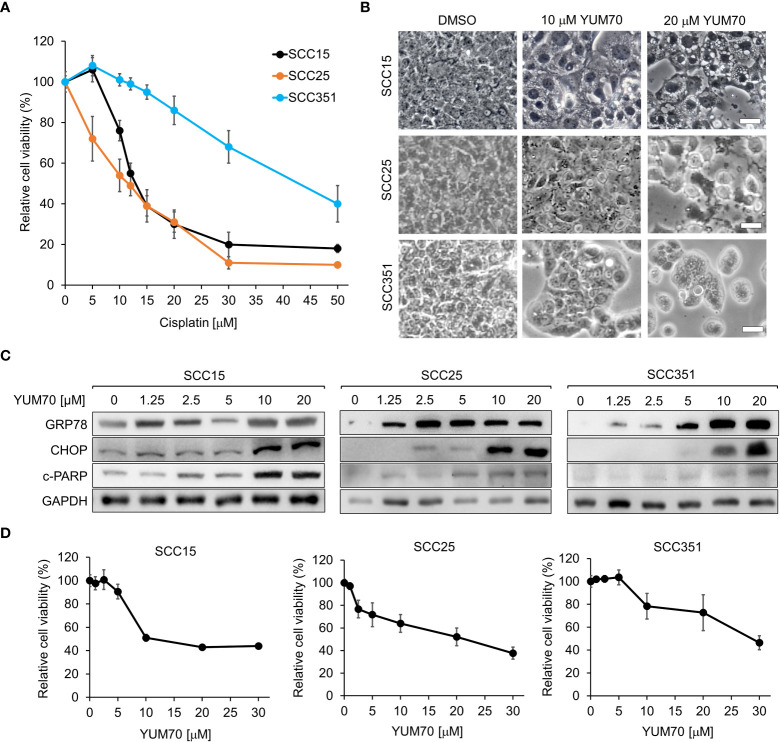
Treatment of GRP78 inhibitor YUM70 induces apoptosis and lowered cell viability of both cisplatin-sensitive and resistant HNSCC cell lines. **(A)** SCC15, SCC25, and SCC351 cells were treated with increasing doses of cisplatin, or PBS as a control, for 48 hr. Cell viability was determined by WST-1 assay. **(B)** Representative images of SCC15, SCC25, and SCC351 cells treated with the indicated concentrations of YUM70, or dimethyl sulfoxide (DMSO) as a control, for 48 hr. Scale bar = 50 μm. **(C)** Western blot analysis of lysates of SCC15, SCC25, and SCC351 cells treated with the indicated concentrations of YUM70 for 48 hr. The protein levels of GRP78, CHOP and cleaved-PARP (c-PARP) were determined, with GAPDH serving as loading control. **(D)** SCC15, SCC25, and SCC351 cells were treated with increasing doses of YUM70 or DMSO for 48 hr. Cell viability was determined by WST-1 assay. Data are presented as means ± S.D. (n=3).

In the second approach, we utilized YUM70 to inhibit GRP78 activity. Compared to SCC15 and SCC25, SCC351, also known as USC-HN-1, is more aggressive and exhibits therapeutic resistance (26). In cell viability assays, SCC351 is more resistant to cisplatin compared to SCC15 and SCC25 (**Figure 3A**). The phase-contrast images of SCC15, SCC25, and SCC351 showed morphological changes, such as cell shrinkage and cell detachment, as well as decrease in cell numbers upon treatment with increasing concentration of YUM70 (**Figure 3B**). For biochemical analysis, the three cell lines were treated with increasing doses of YUM70 ranging from 1.25 to 20 μM for 48 hr. YUM70, *via* inhibition of GRP78 activity, is known to trigger the UPR, as well as increases the expression of GRP78 by increasing the chaperone translation mechanism in pancreatic cancer cells (25). As expected, YUM70 treatment led to general increase in GRP78 protein level. Furthermore, we observed that the levels of apoptotic markers (CHOP, cleaved-PARP) were elevated in all three SCC cell lines in a YUM70 dose-dependent manner (**Figure 3C**). Importantly, even for SCC351 which is highly resistant to the most advanced treatment modalities, YUM70 at 10 and 20 μM was able to induce the apoptotic markers, suggesting that these cells are responsive to YUM70 treatment (**Figure 3C**). Through measurement of the cell viability following YUM70 treatment for 48 hr, we determined that the IC50 for YUM70 for SCC15, SCC25, and SCC351 were 10, 20, and 30 μM, respectively (**Figure 3D**). In different type of cancer cells, the IC50 of YUM70 range was 3-20 μM whereas YUM70 was not toxic at 30 μM for normal pancreatic cells (25).

### Upregulation of GRP78 confers resistance to cisplatin in HNSCC cells

**Figure 4:**
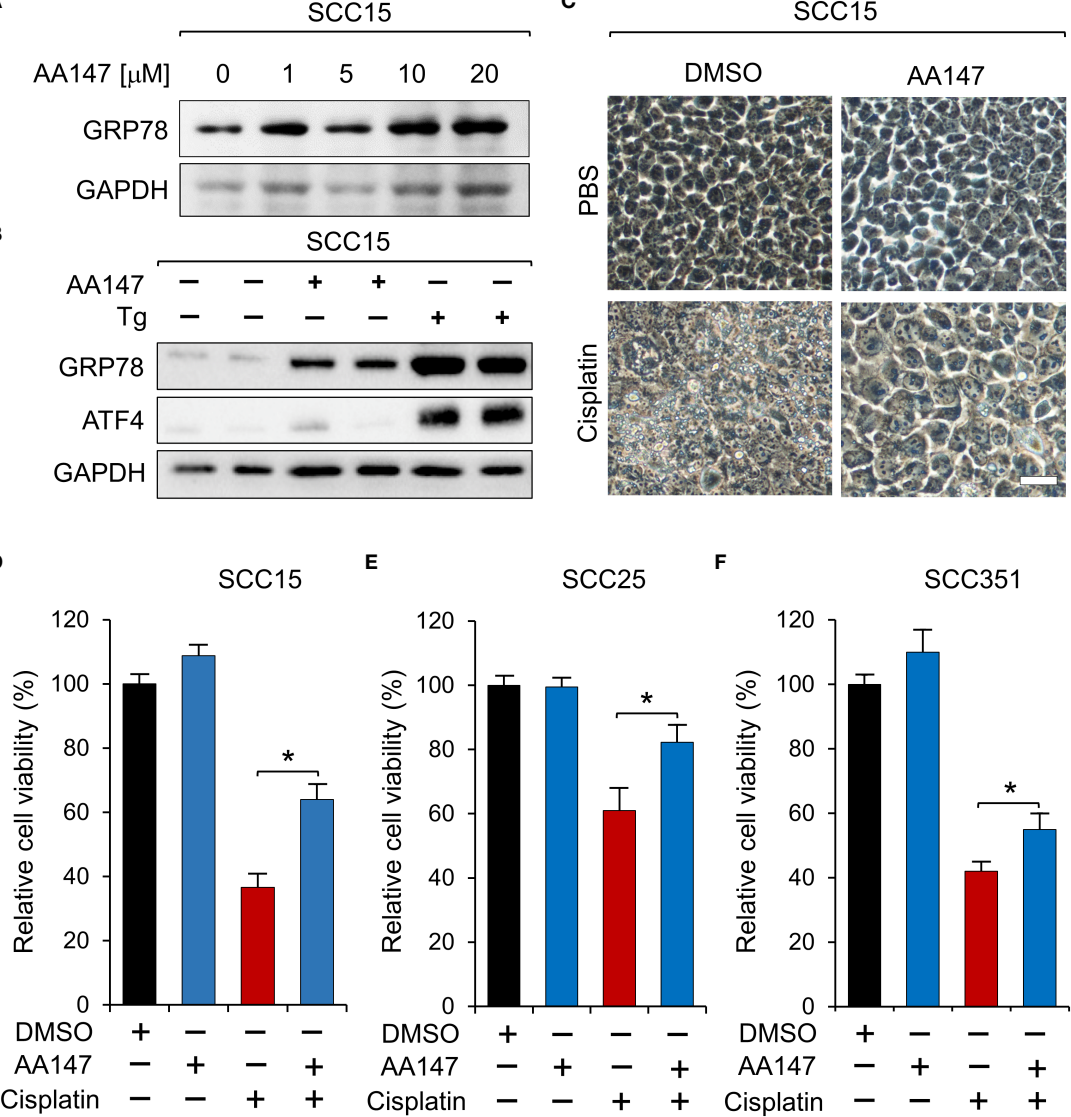
Upregulation of GRP78 attenuates sensitivity to cisplatin in HNSCC cell line. **(A)** SCC15 cells were treated with increasing doses of AA147, or dimethyl sulfoxide (DMSO) as control, for 48 hr and the GRP78 protein level was determined by Western blot, with GAPDH serving as loading control. **(B)** SCC15 cells were treated with either 10 μM AA147 or 300nM of the ER stress-inducer thapsigargin (Tg) for 48 hr. The GRP78 and ATF4 protein levels were determined by Western blot, with GAPDH serving as loading control. **(C)** Representative images of SCC15 cells treated with 12 μM cisplatin or 10 μM AA147 alone or in combination for 48 hr. Scale bar = 50 μm. **(D)** Same as **(C)** except cell viability was determined by WST-1 assay. **(E)** Same as in **(D)** except SCC25 cells were used. **(F)** Same as **(D)** except SCC351 cells were treated with 50 μM cisplatin. Data are presented as means ± S.D. (n=3). *P ≤ 0.05 (Student’s *t* test).

Cisplatin therapy is the frontline and standard treatment for HNSCC; however, development of resistance remains a major challenge. To investigate whether GRP78 contributes to resistance against cisplatin in HNSCC, we treated SCC15 cells with AA147 and observed a dose-dependent increase of GRP78 protein levels (**Figure 4A**). To confirm that AA147 did not activate global UPR, we treated SCC15 cells with AA147, and in parallel, with thapsigargin (Tg), a well-established potent inducer of ER stress. For indication of onset of the UPR, we assayed for the induction of ATF4, which is a predominant UPR marker downstream of the ER stress sensor PERK (35, 36). Thus, Tg treatment strongly induced ATF4 as expected (**Figure 4B**). In contrast, AA147, while capable of inducing GRP78 levels, minimally induced elevation of ATF4 (**Figure 4B**). This demonstrated that AA147 could induce GRP78 without evoking the PERK-mediated UPR branch in SCC15 cells. Next, we treated SCC15 cell with cisplatin alone or in combination with AA147. Live cell imaging revealed that while cisplatin (12μM) treatment led to cell detachment, shrinkage as well as substantial cytoplasmic vacuole formation which is consistent of cell death (37), this was mitigated in cells treated in combination with AA147 (10 μM) (**Figure 4C**). In agreement, cell viability assays showed cisplatin treatment reduced cell viability to about 40%, which was restored to about 60% in combination treatment of AA147 with cisplatin (**Figure 4D**). Similarly, AA147 also partially rescued cisplatin-induced loss of cell viability in SCC25 and SCC351 cells (**Figures 4E, F**). Thus, upregulation of GRP78 by AA147 confers higher resistance to cisplatin in HNSCC cell lines.

### YUM70 re-sensitizes cisplatin resistant SCC15 cells to cisplatin treatment

**Figure 5:**
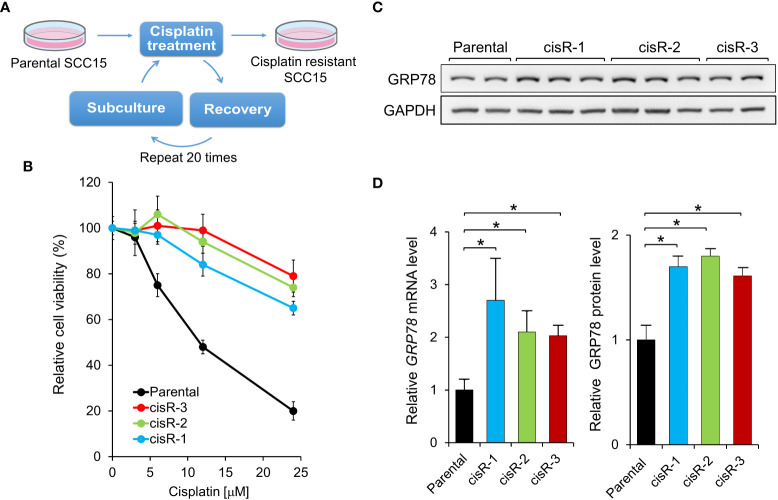
Generation of cisplatin-resistant HNSCC cell lines. **(A)** Schematic drawing for establishing cisplatin-resistant SCC15 cells. The cells were treated step-wise with increasing doses of cisplatin for 48 hr followed by recovery for at least 1 week. Three cisplatin-resistant cell lines, cisR-1, cisR-2, and cisR-3, were generated. **(B)** Parental and the three cisplatin-resistant clones were treated with increasing doses of cisplatin for 48 hr and cell viability was measured by WST-1 assay. **(C)** GRP78 protein expression level was determined by Western blot analysis with GAPDH serving as loading control. **(D)** Left panel: *GRP78* mRNA expression level was determined by quantitative RT-PCR after normalization against *β-actin* mRNA. Right panel: Quantitation of GRP78 protein levels in **(C)** after normalization against GAPDH. Data are presented as means ± S.D. (n=3). *P ≤ 0.05 (Student’s *t* test).

To test whether YUM70 is able to overcome cisplatin resistance in HNSCC, we generated three cisplatin resistant cell lines (cis-R1, cis-R2 and cis-R3) by treating the parental SCC15 cells with cisplatin for 48 hr after which time media was replaced with fresh media and cells were allowed to recover. We repeated the cisplatin incubation, recovery, and passages starting with a low dose of cisplatin and gradually increasing the dose up to 12 μM over the course of 6 months (**Figure 5A**). While the parental SCC15 cells were sensitive to cisplatin in a dosage dependent manner, all three resistant clones were resistant up to 24 μM of cisplatin treatment (**Figure 5B**). Consistent with the above observation that GRP78 upregulation by AA147 protected against cisplatin induced loss of cell viability, following normalization to GAPDH which served as protein loading control, all three cisplatin resistant cell lines exhibited elevated levels of GRP78 protein compared to the sensitive parental cells (**Figures 5C, D**). Correspondingly, *GRP78* mRNA levels were also elevated in all three resistant cell lines (**Figure 5D**). Since cis-R3 exhibits most resistance against cisplatin among the 3 resistant cell lines, it was chosen for the subsequent analyses.

**Figure 6:**
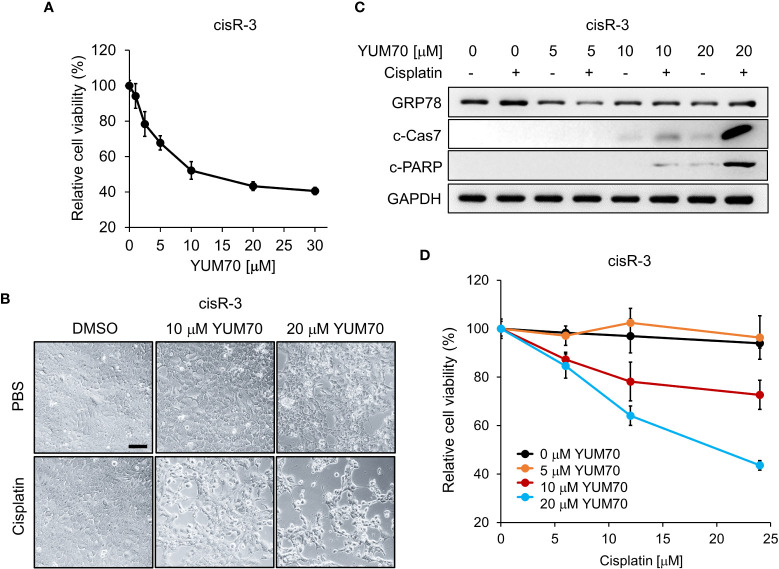
YUM70 reduces cell viability and restores cisplatin sensitivity in cisplatin-resistant SCC15. **(A)** Cisplatin-resistant SCC15 cells (the cisR-3 line) were treated with increasing doses of YUM70 for 48 hr. Cell viability was determined by WST-1 assay. DMSO was used as vehicle control. **(B)** Representative images of cisR-3 cells treated with the indicated dosages of YUM70 alone or in combination with 12 μM cisplatin for 48 hr. Scale bar = 100 μm. **(C)** Western blot of lysates of cisR-3 cells treated with the indicated dosages of YUM70 for 48 hr, alone or in combination with 12 μM cisplatin. The protein levels of cleaved Caspase 7 (c-Cas7) and cleaved-PARP (c-PARP) were measured, with GAPDH serving as loading control. **(D)** Cell viability of cisR-3 cells treated with the indicated dosages of YUM70, alone or in combination with the indicated dosages of cisplatin for 48 hr was measured by WST-1 assay. Data are presented as means ± S.D. (n=3).

First, we observed that the viability of cis-R3 decreased in a dose-dependent manner with increasing amounts of YUM70, with an IC50 of 10μM (**Figure 6A**). In agreement, live cell images of cis-R3 treated with increasing doses of YUM70 showed an increase of vacuole structures and a decrease in cell density in the treated cells, and with combination treatment with cisplatin, further decrease in cell numbers was observed (**Figure 6B**). Western blot analysis of the cell lysates showed that combination of YUM70 with cisplatin led to onset of apoptotic markers cleaved Caspase-7 and cleaved PARP (**Figure 6C**). Caspase-7 is localized to the outer membrane of the ER and is kept in inactive form through complex formation with GRP78; upon release from GRP78, Caspase-7 is cleaved into its active form (38). In the absence of YUM70, the viability of cis-R3 cells was unaffected up to 24 μM cisplatin (**Figure 6D**). However, when YUM70 was used in combination with cisplatin, the cell viability of cisR-3 decreased as the concentration of cisplatin increased, suggesting cisR-3 became re-sensitized to cisplatin treatment (**Figure 6D**). Collectively, these results indicate that YUM70 is capable of re-sensitizing the resistant cells to cisplatin therapy.

### Co-treatment of YUM70 and cisplatin reduced clonogenicity in cisplatin resistant HNSCC

**Figure 7:**
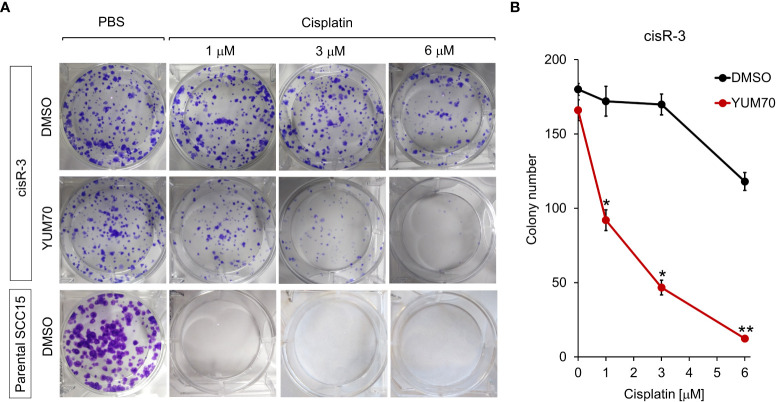
YUM70 in combination with cisplatin reduces clonogenic capacity of cisplatin-resistant SCC15 cells. **(A)** CisR-3 cells or parental SCC15 cells were treated with the indicated dosages of cisplatin alone or in combination with 2.5 μM of YUM70, with DMSO serving as vehicle control, for 48 hr. Colonies formed after 12 days post-treatment were stained with crystal violet and counted. **(B)** Quantitative results of **(A)**. Data are presented as means ± S.D. (n=3). *P ≤ 0.05, **P ≤ 0.01 (Student’s *t* test).

To further investigate if YUM70 treatment could re-sensitize cisR-3 cells to cisplatin treatment, clonogenic colony formation assay was performed. As expected, cisplatin treatment at 1, 3, and 6 μM for 48 hr in the parental SCC15 cells resulted in a complete loss of clonogenicity (**Figure 7A**). For cisR-3, the clonogenic ability was unaffected when treated alone with 1μM cisplatin, 3 μM cisplatin, or 2.5 μM YUM70, while 6 μM cisplatin treatment alone reduced colony number by 30% (**Figures 7A, B**). However, in a dosage dependent manner, cisplatin treatment in conjunction with 2.5 μM of YUM70 for 48 hr significantly reduced clonogenicity in cisR-3 cells (**Figures 7A, B**).

### Co-treatment of YUM70 and cisplatin induced apoptosis in 3D spheroids of cisplatin-resistant HNSCC

**Figure 8:**
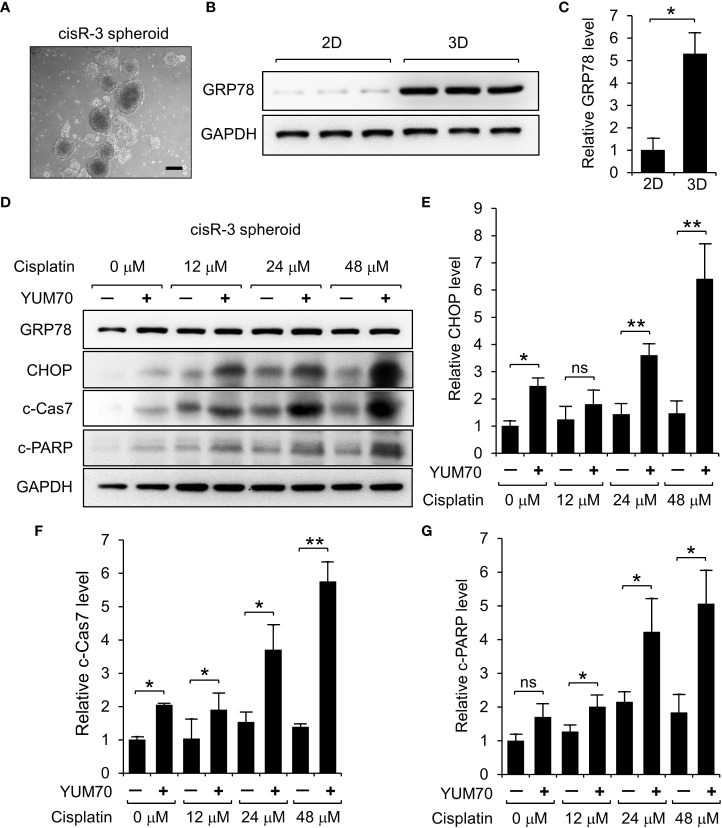
YUM70 treatment induces apoptosis in cisplatin-treated cisR-3 spheroids. **(A)** A representative image of cisR-3 spheroids. Scale bar = 200 μm. **(B)** GRP78 protein levels in cisR-3 grown in 2D culture and 3D spheroid culture was determined by Western blot with GAPDH serving as loading control. **(C)** Quantitative results of **(B)**. **(D)** Western blot analysis of lysates of cisR-3 spheroids treated with the indicated dosages of cisplatin for 48 hr, alone or in combination with 10 μM YUM70. The protein levels of GRP78, CHOP, cleaved Caspase 7 (c-Cas7), and cleaved-PARP (c-PARP) were determined with GAPDH serving as loading control. **(E-G)** Quantitative results of **(D)** after normalization against the GADPH level. Data are presented as means ± S.D. (n=3). *P ≤ 0.05, **P ≤ 0.01, ns, not significant (Student’s *t* test).

To further investigate whether YUM70 can enhance cisplatin sensitivity to HNSCC cells in a physiologically relevant condition, we grew 3D spheroids using cisR-3 cells (**Figure 8A**). Western blot results show that GRP78 level was upregulated in cisR-3 spheroids compared to the cisR-3 cells grew in 2D monolayer culture (**Figures 8B, C**). Next, the spheroids were treated with cisplatin with or without YUM70 for 48 hr and the levels of CHOP, cleaved Caspase-7 (c-Cas7) and cleaved PARP (c-PARP) were determined. Cisplatin at 12 or 24 μM did not cause significant changes in the 3 apoptotic markers while YUM70 treatment alone was able to induce CHOP and c-Cas7 cleavage (**Figures 8D–G**). Importantly, all three apoptotic markers increased dramatically as the doses of cisplatin increased in combination with YUM70 (**Figures 8D–G**). These results demonstrated that YUM70 treatment re-sensitized cisplatin-resistant HNSCC cells to cisplatin.

## Discussion

While ER stress has been strongly associated with cancer cell survival and chemoresistance, including HNSCC, therapeutic agents exploiting the ER stress mediated UPR as anti-cancer treatments are just emerging (35). In this study, we focused on targeting GRP78, a key pro-survival component for the UPR and uncover the following interesting findings.

First, our independent analysis of the transcript expression levels of *GRP78* in HNSCC, based on general as well as specific cancer subtypes, showed that *GRP78* transcripts is uniformly elevated in the primary HNSCC tumors compared to normal tissues and that high transcript level of *GRP78* is associated with poor prognosis. Our results validate previous studies that clinically, high GRP78 expression, at both protein and mRNA levels, is associated with worse patient survival (39–41). Utilizing two independent siRNAs targeting different regions of human *GRP78*, we established that knockdown of GRP78 led to dramatic induction of CHOP, a UPR apoptotic marker in HNSCC, thus GRP78 integrity is a critical anti-apoptotic protein in HNSCC.

Taking advantage of the discovery of YUM70, a small molecule inhibitor of GRP78 which binds directly to GRP78 and inhibits its enzymatic activity, we showed that YUM70 in clinically relevant dosages previously established in cell culture and animal models with minimal toxicity (25), mimics GRP78 knockdown and led to robust CHOP and c-PARP induction. YUM70-mediated inhibition of GRP78 activity could lead to an increased ER stress and UPR target gene induction. Consequently, YUM70 treatment causes ER-stress mediated apoptosis by PERK-ATF4-CHOP induction. It is important to note that while YUM70 also increases the expression of GRP78 by increasing the chaperone translation mechanism, all newly synthesized GRP78 will be continuously subjected to the inhibitory activity YUM70, thus negating any pro-survival effects of GRP78 in the ER stressed cells. Importantly, the induction of apoptosis by YUM70 is not only observed in SCC15 and SCC25 HNSCCs, but also in SCC351 cells which exhibit resistance to multiple therapeutic agents, including cisplatin. This result raises the interesting possibility that YUM70 may be able to reverse cisplatin resistance developed in HNSCC during the course of cisplatin treatment.

To test this, we subjected SCC15 cells to multiple rounds of cisplatin treatment and isolated several cisplatin-resistant clones. Consistent with the notion that GRP78 confers chemoresistance to HNSCC, both mRNA and protein levels of GRP78 were elevated in these clonal lines. While these cells exhibited resistance to cisplatin up to 24 μM, in combination of YUM70, cell viability was considerably reduced in a YUM70 dosage dependent manner. Correspondingly, we observed that under combination treatment of YUM70 and cisplatin, robust cleavage of Caspase-7 was observed. As part of the apoptotic machinery, Caspase-7 is uniquely located at the outer ER membrane and bound to GRP78 which maintains it as an inactive form (38). Upon ER stress, Caspase-7 is released from GRP78 and becomes activated upon cleavage. The general apoptotic marker c-PARP is also activated. Thus, inhibiting GRP78 by YUM70 is able to rescue acquired cisplatin resistance of HNSCC. We further demonstrated in the context of colony survival assays of the resistant clonal cells, cisplatin enhances YUM70 mediated suppression of cell viability in a dosage dependent manner.

3D cancer models are known to recapitulate biological features of the original cancer and thus have better physiological relevance for understanding diseases and testing therapeutics (42–44). In our 3D spheroid culture model, both UPR-mediated apoptotic markers (CHOP and c-Cas7) were strongly induced following YUM70 and cisplatin combination treatment and c-PARP levels also dramatically increased as the dose of cisplatin increases in conjunction with YUM70. Due to YUM70’s ability to inhibit GRP78 with high specificity, induce apoptosis in cancer cells but not in normal cells (25), and reverse chemotherapy resistance, YUM70 holds great promise for future clinical development which includes further improvements in potency, safety and bioavailability. In conclusion, small molecule GRP78 inhibitors such as YUM70 represent invaluable tools to suppress GRP78 activity. Its applications in HNSCC therapy and other types of cancer that depend on GRP78 function warrant vigorous investigation.

## Data availability statement

The original contributions presented in the study are included in the article/supplementary material. Further inquiries can be directed to the corresponding author.

## Author contributions

VY and AL conceptualization; V.Y and BW investigation and data acquisition; VY, B.W and AL formal analysis; VY, BW and AL writing manuscript; VY, BW and AL review manuscript; AL funding acquisition. All authors contributed to the article and approved the submitted version.
